# Recovery of Zinc from Metallurgical Slag and Dust by Ammonium Acetate Using Response Surface Methodology

**DOI:** 10.3390/ma16145132

**Published:** 2023-07-20

**Authors:** Xuemei Zheng, Jinjing Li, Aiyuan Ma, Bingguo Liu

**Affiliations:** 1 School of Chemistry and Materials Engineering, Liupanshui Normal University, Liupanshui 553004, China; zxm_lpssy19@163.com (X.Z.); 19985189154@163.com (J.L.); 2 Key Laboratory of Unconventional Metallurgy, Faculty of Metallurgical and Energy Engineering, Kunming University of Science and Technology, Kunming 650093, China

**Keywords:** metallurgical slag and dust, zinc recovery, ammonium acetate, response surface methodology

## Abstract

Metallurgical slag and dust (MSD) are abundant Zn-containing secondary resources that can partially alleviate the shortage of zinc minerals, with hazardous characteristics and a high recycling value. In this work, the process conditions of recycling Zn from MSD materials leaching by ammonium acetate (NH_3_-CH_3_COONH_4_-H_2_O) were optimised using response surface methodology (RSM). The influences of liquid/solid ratio, stirring speed, leaching time, total ammonia concentration, and the interactions between these variables on the Zn effective extraction rate during the ammonium acetate leaching process were investigated. Additionally, the predicted regression equation between the Zn effective extraction rate and the four affecting factors was established, and the optimal process parameters were determined with a stirring speed of 345 r/min, leaching temperature of 25 °C, [NH_3_]/[NH_4_]^+^ of 1:1, total ammonia concentration of 4.8 mol/L, liquid/solid ratio of 4.3:1, and leaching time of 46 min. The Zn effective extraction rates predicted by the proposed model and the measured values were 85.25% and 84.67%, respectively, with a relative error of 0.58% between the two values, indicating the accuracy and reliability of the proposed model. XRD and SEM-EDS analysis results showed that Zn_2_SiO_4_, ZnS, and ZnFe_2_O_4_ were among the main factors affecting the low extraction rate of zinc from metallurgical slag dust. This work established a new technology prototype for the effective and clean extraction of zinc resources, which can provide new routes to effectively utilise Zn-containing MSD materials and lay a foundation for developing other novel techniques for recycling Zn from Zn-containing secondary resources.

## 1. Introduction

Zinc (Zn) is an essential metal in modern life, and its main mineral source is sphalerite (ZnS). Zinc has a low melting point, good melt fluidity, and is easy to die-cast; hence, it is frequently utilised to produce precision castings [1]. Zinc is a negatively charged metal, and its electrode potential is more negative than iron (Fe), with the result that zinc can be corroded instead of iron through electrochemical action [2,3]. Therefore, zinc is also widely applied as battery anode materials and plated steel materials. With the increasing demand for galvanised materials in the battery industry, automobile industry, and construction industry, the mining volume of sphalerite has increased, and the ore grade has declined year by year [4]. The ever-increasing demand for galvanised materials and the scarcity of zinc resources have forced the development and utilisation of other zinc-containing hazardous waste resources, such as metallurgical slag and dust (MSD).

MSD materials source from the smelting process of steel, zinc (Zn), lead (Pb), and copper (Cu), etc., and their production, is rising sharply [5,6,7]. As hazardous materials, MSD materials are rich in heavy metals like Zn, Cu, and Fe, as well as toxic components such as As, Hg, Pb, Cr, and Cd [8,9]. Hence, simple burial and stockpile disposal for MSD materials are inadvisable. In addition, once the content of zinc and other elements in metallurgical slag exceeds the specified value, it will damage the refractory materials of the furnace cavity, further shortening the service life of smelting furnace, and cause the productivity of smelting furnace to decrease, as well as leading to operation difficulties [10,11]. Therefore, the research and development on the clean, efficient, and economical approaches for recycling MSD materials have good environmental and practical significance; moreover, the relevant metallurgical industry will gain considerable added value and economic benefits based on the developed approaches.

Metallurgical slag and dust have a high recycling value andcontain diverse conventional smelting metals like Zn, Fe, and Pb, and precious metals like Ag, Au, and in [12,13]. However, MSD materials have diverse compositions, wherein Zn mainly presents as ZnO, frantzite, and zinc silicate; Fe mainly presents as Fe_3_O_4_ and frankite; and Ca mainly presents as CaCO_3_. Further, Fe, Zn, and Ca also exist in the form of silicate [14,15,16]. Furthermore, the structures of MSD materials are complicated, with different metal oxides, chlorides, carbon-containing compounds, and gangues doped and wrapped together. The diverse compositions and complicated structures of MSD materials make the recycling process difficult [17,18]. At present, the widely applied method for zinc extraction is a hydrometallurgy leaching approach with sulfuric acid as the leaching agent, which consumes less energy than pyrometallurgy methods [19]. However, Zn-containing MSD contains zinc by-products with various impurities, including Fe (up to 14%), Ca (up to 19%), Cl (up to 12%), and F (up to 2%) [20,21]. Moreover, the high-content components like Fe, Cl, Ca, and gangues in the zinc-containing MSD materials, consume excessive acid and complicate the purification process become [22]. The application of zinc extraction by conventional acid methods is limited by the disadvantages of long purification time, large acid consumption, high energy consumption, low-quality electrolytic zinc, and low recovery rate [23,24,25]. Therefore, alkaline leaching is gradually proposed for zinc extraction. In the literature, applications of ammonium salts such as ammonium chloride, ammonium sulphate, and ammonium bicarbonate have been reported on the Zn extraction from single-phase zinc-containing minerals (e.g., ZnO, smithsonite, and hydrozincite), and the effects of zinc extraction are sound [26,27,28]. The essence of the alkaline leaching method is that ammonia compounds can form tetraammine zinc ion ([Zn(NH_3_)_4_]^2+^) coordination compounds with zinc metal (Zn^2+^) ions to prevent impurities containing Fe, Al, and Si from entering the leaching solution, thereby achieving the effective separation of Zn and impurities [29,30]. Additionally, Rao et al. [31] highlighted that the zinc effective extraction rate leaching using a mixed solution of NH_4_Cl-NH_3_-NTA was higher than that using single NH_4_Cl-NH_3_ solution leaching. The addition of nitrosotriacetic acid (N(CH_2_COOH)_3_, i.e., NTA) promotes the transformation of [Zn(NH_3_)_4_]^2+^ and [Zn(NTA)_2_]^4−^ coordination compounds into more stable [Zn(NTA)(NH_3_)_2_]^−^ coordination compounds, thereby effectively improving the zinc effective leaching rate [31]. Therefore, ammonium ion (NH^4+^) and carboxylate anion (RCOO^−^) play crucial and complementary roles in the zinc leaching process. Compared with a single ammonia leaching method, the leaching solution mixed with NH^4+^ ion and RCOO^−^ anion contributes to extracting zinc from MSD materials more efficiently. Therefore, the leaching solution mixed with NH^4+^ ions and RCOO^−^ anions can be considered for introduction into the leaching process of zinc-containing metallurgical slag and dust with complex structures and diverse components.

In this work, response surface methodology (RSM) [32,33,34] was introduced into the process optimisation of recovering Zn from Zn-containing MSD materials by coordination leaching using NH_3_-CH_3_COONH_4_-H_2_O solution. The influences of liquid/solid ratio, stirring speed, leaching time, total ammonia concentration, and the interactions between them on the Zn effective extraction rate were explored using the central composite design (CCD) of RSM, and a mathematic model of the factors affecting the Zn effective extraction rate was established. Additionally, the fitting analysis, confidence analysis, and variance analysis of the regression equation of the proposed model; the linear correlation between the experimental and predicted values of the Zn effective extraction rate; and the normal probability characteristics of residuals for the Zn effective extraction rate were systematically investigated to confirm the credibility and accuracy of the proposed model. Moreover, the accuracy of the optimisation parameters obtained by RSM was verified through comparing the predicted value and the average value of the Zn effective extraction rate, as determined by three parallel experiments.

## 2. Materials and Methods

### 2.1. Chemical Composition of MSD Materials

The metallurgical slag and dust (MSD) materials studied in this work were drawn from a local enterprise located in Yunnan province (Qujing, China), which is mainly engaged in the recovery and utilization of zinc secondary resources. The MSD sample is a mixture of various MSD materials. After completely drying at 85 °C until no further mass loss was observed, the composition analysis of the MSD sample was determined by the ICP method using Agilent 5110 (OES, Agilent Technologies, Santa Clara, CA, USA), and the analytical results are displayed in Table 1. As determined in Table 1, the MSD sample has a complex composition: the contents of Zn, Fe, Ca, and Cl were high, gangue, and scattered In were observed. Therefore, the MSD sample exhibits a high recycling value.

### 2.2. Particle Size Distribution of MSD Materials

**Figure 1:**
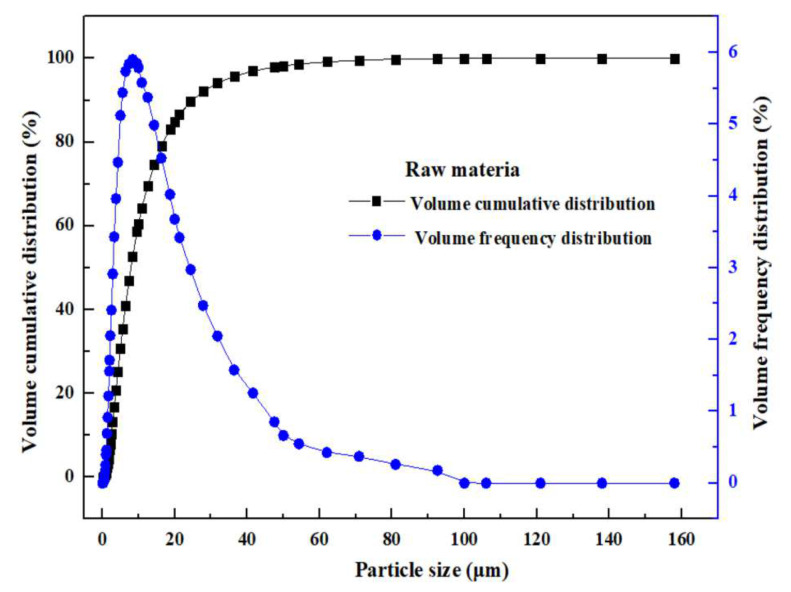
Particle size distribution of the MSD sample.

Figure 1 shows the particle size distribution of the MSD samples. Table 2 presents the particle size values at the particle size level of D10, D50, D90, and D98 of the MSD samples, together with the volume average particle size, area average particle size, and corresponding surface area to volume ratio for the MSD sample. Table 2 shows that 90% of MSD samples have a particle size of 24.85 μm.

### 2.3. Experimental Design of Response Surfaces and Leaching Experimental Methods

#### 2.3.1. Experimental Design of Response Surfaces

In this study, the stirring speed (*X*_1_, r/min), leaching time (*X*_2_, min), total ammonia concentration (*X*_3_, mol/L), and liquid/solid ratio (*X*_4_, mL/g) were selected as the variables in the leaching experiments. The Zn effective extraction rate by the coordination leaching process using NH_3_-CH_3_COONH_4_-H_2_O solution was defined as the response value (*Y*, %). The leaching experimental conditions were provided with the molar ratio of [NH_3_]/[NH_4_]^+^ as 1:1, and the leaching temperature as 25 °C.

Table 3 summarises the codes of the centre combination design (CCD) optimisation based on RSM, with four factors and three levels. In the centre combination design (CCD), each factor has 2^4^ sufficient factorials, including eight factorial points, eight axial points, and six repeated centre points. The full-factor centre was optimised over 30 experiments to identify the optimised values of the dependent variable and the independent variables. The number (i.e., 30) of experiments was calculated using the following formula:

N = 2^n^ + 2n + n_c_ = 2^4^ + 2 × 4 + 6 = 30 (1)

where N denotes the number of the needed experiments, n presents the number of factors, and n_c_ indicates the number of repeated centre points.

During response surface optimisation, the model accuracy was verified before data analysis. The accuracy of the proposed model was investigated using Design Expert (STAT-EASE, Stat Ease Inc., Minneapolis, MN, USA). The Zn effective extraction rate (*Y*, %) was the dependent variable, and the stirring speed (*X*_1_, r/min), leaching time (*X*_2_, min), total ammonia concentration (*X*_3_, mol/L), and liquid/solid ratio (*X*_4_, mL/g) were selected as the variables.

#### 2.3.2. Leaching Experimental Methods

Before the XRD and SEM analysis, the MSD samples were dried at 85 °C until no further mass loss was observed. Then, 20.00 g of the dried MSD material was sampled and mixed with the freshly prepared ammonium acetate (NH_3_-CH_3_COONH_4_-H_2_O) leaching agent in a 300 mL conical flask with stirring. The detailed experimental parameters for leaching zinc from the MSD sample were as follows: the leaching time was set from 5 min to 65 min; the stirring speed was controlled between 200 r/min and 400 r/min; the total ammonia concentration was set between 2 mol/L and 6 mol/L; the liquid/solid ratio was adjustable from 2 mL/g to 6 mL/g, and the leaching temperature was 25 °C. This leaching process was performed in a thermostatic water bath. After leaching, solids and liquids were separated, and the Zn concentration in the leaching solution was measured by EDTA titration. The Zn effective extraction rate (*η*_Zn_, %) was determined by Equation (2):

(2) $$\eta_{Zn}=\frac{C_{Zn}\times V}{m\times \omega_{Zn}}\times 100\%$$

In Equation (1), *C*_Zn_ indicates the Zn concentration in the leaching solution, g/L; *V* denotes the volume of the leaching solution, L; *m* presents the MSD sample mass, g; and *w*_Zn_ denotes the mass percent of zinc in the MSD sample, 24.74%.

After obtaining the Zn effective extraction rates under different leaching conditions using the above calculation, response surface methodology (RSM) was introduced into the process optimisation of recycling Zn from Zn-containing MSD materials by coordination leaching using NH_3_-CH_3_COONH_4_-H_2_O solution.

### 2.4. Leaching Reaction of Zinc Extraction Process

For the leaching system of ZnO-NH_3_-CH_3_COONH_4_-H_2_O, the dissolved zinc oxide (ZnO) can combine with ammonium ions (NH_4_^+^) and ammonia (NH_3_) to form soluble [Zn(NH_3_)_i_]^2+^ complexes, as shown in Equations (3) and (4). In addition, zinc ions can combine with carboxylate anion (RCOO^−^) to form stability complexes, as shown in Equation (5).

ZnO + iNH_4_^+^ = [Zn(NH_3_)_i_]^2+^ + H_2_O+ (i − 2)H^+^ (3)

ZnO + iNH_3_ + H_2_O = Zn(NH_3_)_i_^2+^ + 2OH^−^ (4)

(5) $$2\mathrm{RCO}O^{-}+\mathrm{ZnO}\overset{\;(H_{3}O^{+})\;}{↔}(\mathrm{RCOO}{)}_{2}\mathrm{Zn}+H_{2}O$$

## 3. Results and Discussion

### 3.1. Results of the Optimization of Response Surfaces

To reduce the system errors during the NH_3_-CH_3_COONH_4_-H_2_O leaching process, the sequence of experiments was determined randomly by Design Expert (STAT-EASE, USA), and the central composite experimental design and corresponding zinc effective leaching rates are illustrated in Table 4.

### 3.2. Model Verification and Statistical Analysis

The secondary polynomial regression equation of the Zn effective extraction rate by coordination leaching from the MSD sample using NH_3_-CH_3_COONH_4_-H_2_O solution was obtained using the least-squares method, as follows:

*Y* = 79.19 + 0.38*X*_1_ + 0.84*X*_2_ + 8.55*X*_3_ + 7.25*X*_4_ − 0.012*X*_1_*X*_2_ + 0.10*X*_1_*X*_3_ + 0.28*X*_1_*X*_4_ − 0.20*X*_2_*X*_3_ + 0.36*X*_2_*X*_4_ − 4.30*X*_3_*X*_4_ − 0.16*X*_1_^2^ − 0.69*X*_2_^2^ − 3.27*X*_3_^2^ − 3.34*X*_4_^2^ (6)

The model accuracy can be further demonstrated using a variance analysis to identify the significances of all factors in the polynomial equation, as well as to judge the effectiveness of the model. Table 5, Table 6 and Table 7 indicate the fitting analysis, confidence analysis, and variance analysis for the regression equation of the proposed model, respectively.

Table 5 displays the numerical analysis of the experimental response surface. During the CCD of the response surface, the value of (Prob > *F*) of the high-accuracy regression model should be lower than 0.05, in order to guarantee an effective simulation. Moreover, the value of (Prob > *F*) is required to exceed 0.05, which denotes a high fitting degree of the regression equation. As depicted in Table 4, the quadratic model had a Prob > *F* value of below 0.0001 and an anomalistic term of 0.0674, demonstrating that the designed model had a significant fitting effect. Hence, the quadratic model was used as the fitting model for the centre combination design (CCD).

The applicability and accuracy of the designed model are indicated by its correlation coefficient (*R*^2^). Table 6 presents the credibility analysis results of the zinc effective leaching rate. As presented in Table 6, the *R*^2^ value of the quadratic model was 0.9951, demonstrating that the model presented a good fit effect with the experimental data. Generally, the difference between the predicted *R*^2^ and calibrated *R*^2^ values should be below 0.2. In this case, the *R*^2^_Pred_ value was 0.9715 and the *R*^2^_adj_ value 0.9902, suggesting that the proposed model can accurately predict the experimental data. In addition, Adeq precision can reflect the signal-to-noise ratio, and Adeq precision > 4 indicates a reasonable signal-to-noise ratio. In this case, Adeq precision was determined at 44.882 (Table 6), indicating a high signal-to-noise ratio. Furthermore, according to the MYERS theory, the correlation coefficient of a model should be higher than 0.8, where the specific value indicates good fitting performance. In this study, *R*^2^ = 0.9951, *R*^2^_adj_ = 0.9902, and *R*^2^_Pred_ = 0.9715 demonstrated a good fitting performance of the proposed model. Therefore, it can be surmised from the above analysis that the proposed model is applicable to the examined case.

Table 7 depicts the variance analysis results of the response surface quadratic model. As illustrated in Table 7, the *F* value of the proposed model was 215.67. The probability that the signal-to-noise ratio is exposed to error was 0.01% (Prob > *F* < 0.0001), indicating a high accuracy and good fitting performance of the proposed regression model. The Prob > *F* value of a variable less than 0.05 indicates that the variable significantly affects the response value. Among all the listed affecting factors, *X*_2_, *X*_3_, *X*_4_, *X*_3_*X*_4_, *X*_2_^2^, *X*_3_^2^, and *X*_4_^2^ had significant effects on the Zn effective extraction rate using NH_3_-CH_3_COONH_4_-H_2_O leaching. The variance analysis demonstrated that the proposed model showed a good fit to the experimental data, and the model can accurately predict the Zn effective extraction rate achieved by coordination leaching from MSD using NH_3_-CH_3_COONH_4_-H_2_O.

**Figure 2:**
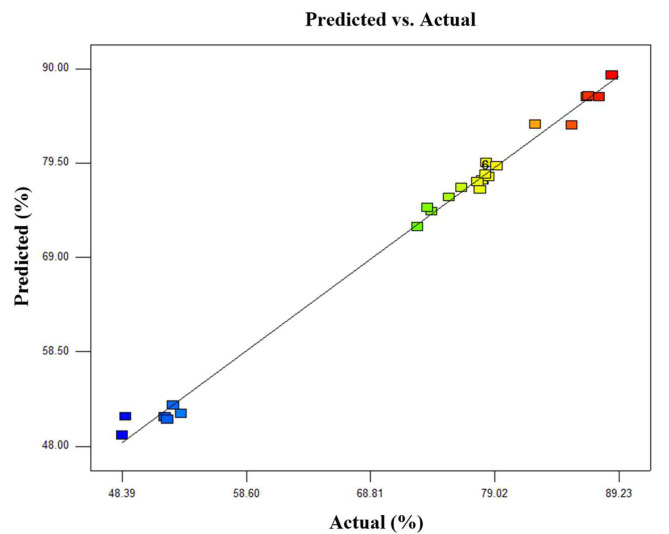
Linear correlation between the experimental and predicted values of Zn effective leaching rate.

Figure 2 displays the linear correlation between the predicted and experimental values of the Zn effective extraction rate. It can be concluded from Figure 2 that the experimental values were highly consistent with the predicted values. The experimental values were uniformly scattered on both sides of the predicted values, demonstrating that the quadratic model was suitable to describe the correlation of experimental factors and the Zn effective extraction rate. In other words, the proposed model accurately reflected correlations between different parameters.

**Figure 3:**
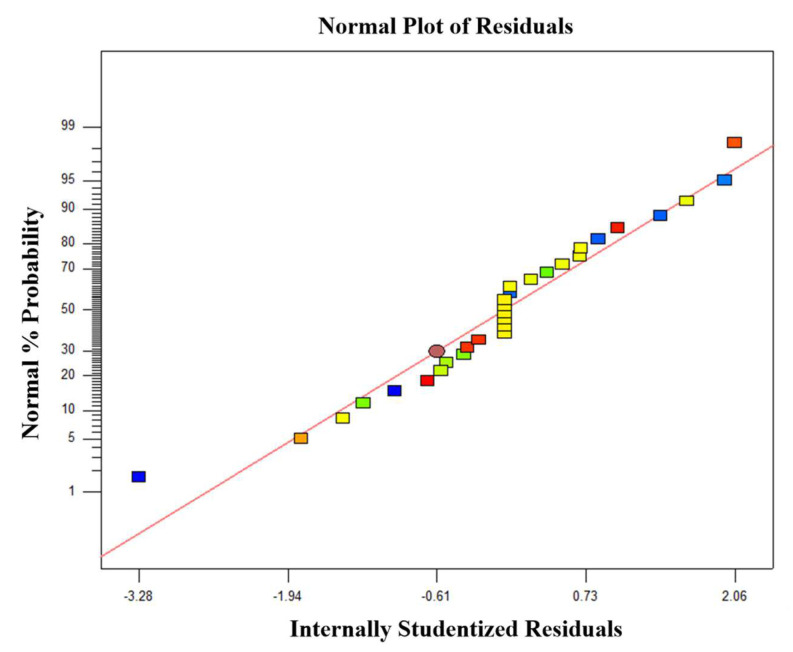
Normal probability plot of studentized Zn effective extraction rate.

Figure 3 shows the normal probability plot of the residuals for the Zn effective extraction rate. Herein, the division of normal probability on the *Y*-axis reflects a normal distribution of residuals. As displayed in Figure 3, the residuals of the Zn effective extraction rate are distributed along a straight line, demonstrating a normal distribution of the experimental residuals. The residuals on the *X*-axis reflect the differences between the experimental responses and the predicted values by the model. The residuals are concentrated in the middle region in an S-shaped curve, suggesting a good accuracy of the proposed model.

### 3.3. Analysis of the Response Surface Model

**Figure 4:**
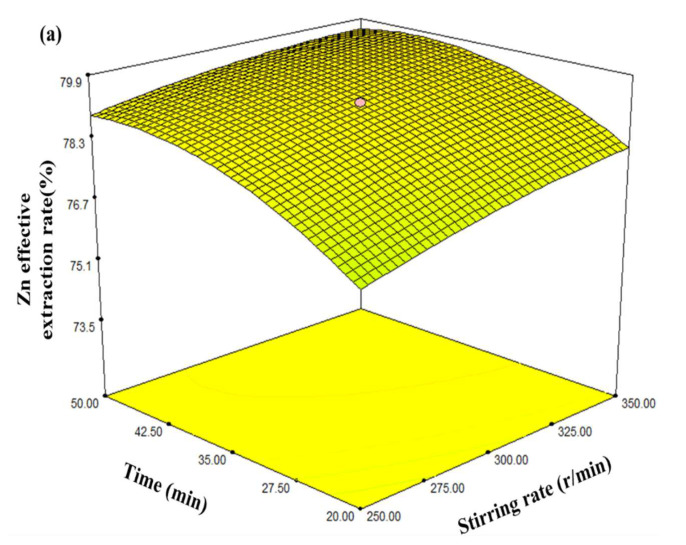

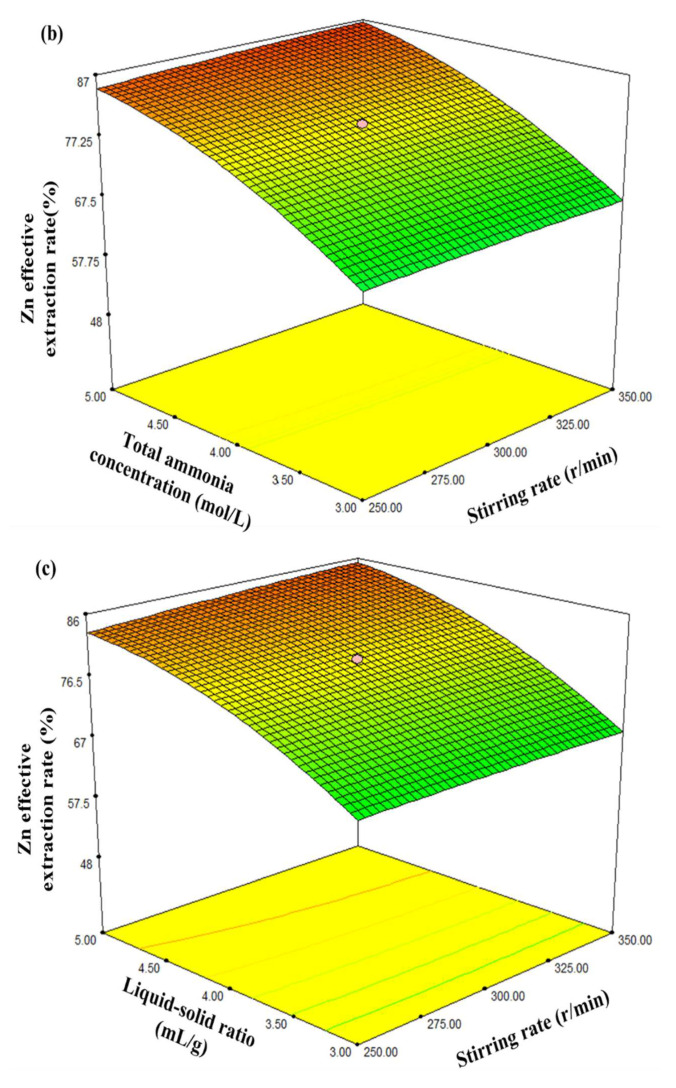

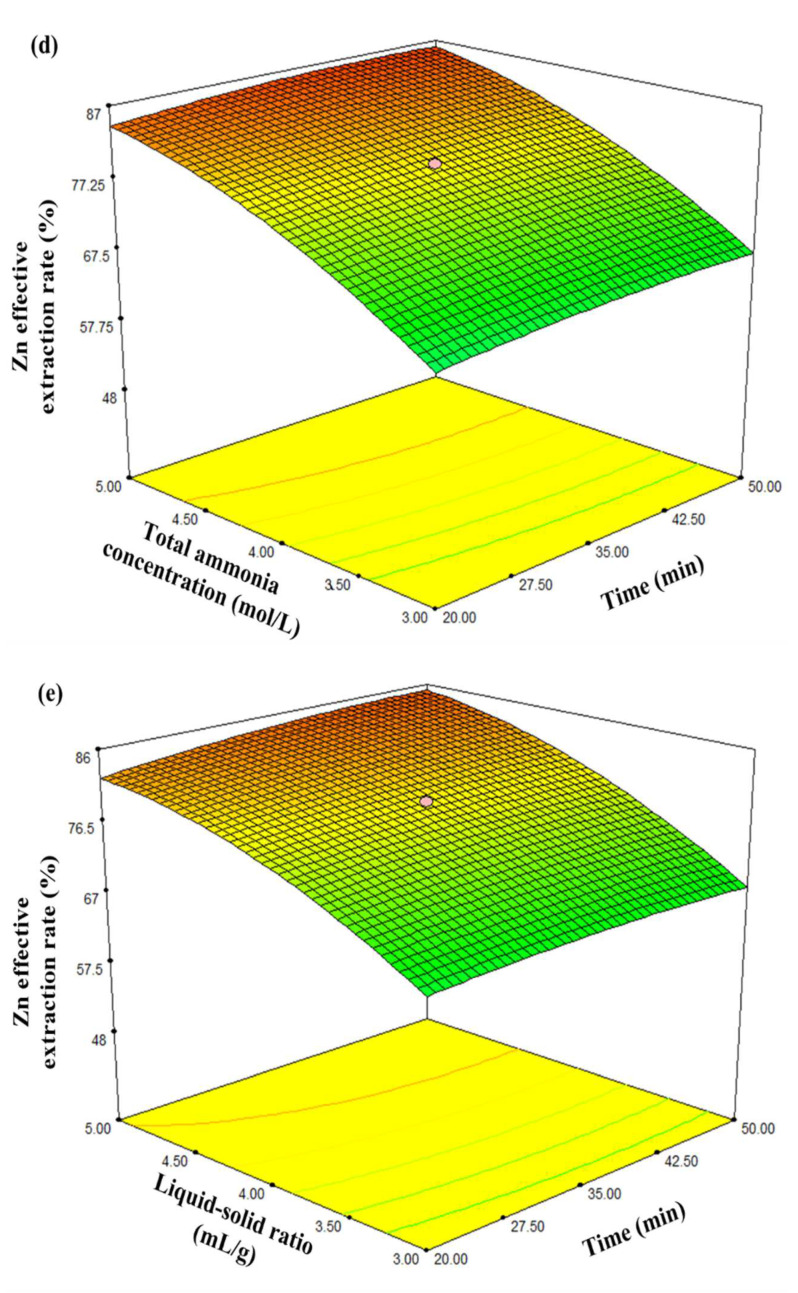

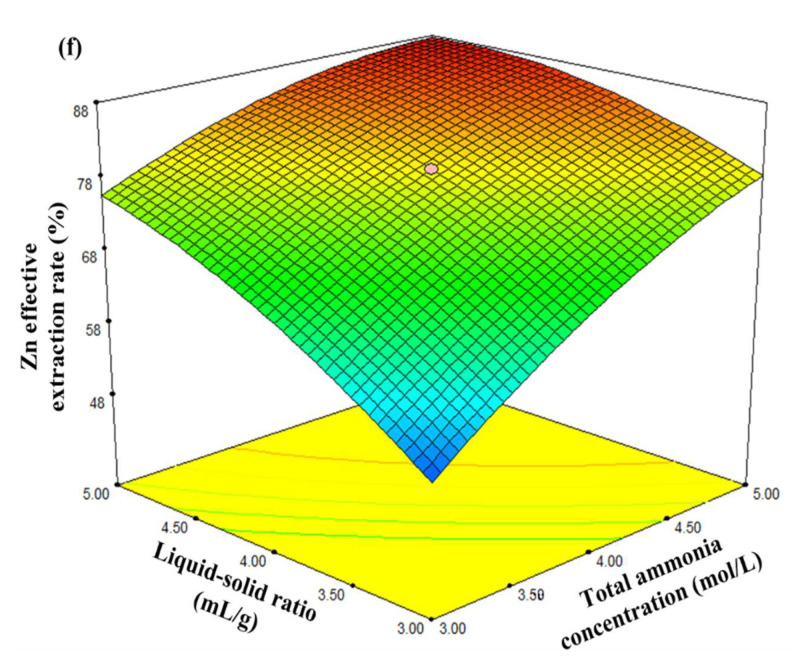
Response surface plots for stirring speed vs. leaching time vs. total ammonia concentration vs. liquid/solid ratio, (**a**) leaching time vs. stirring speed; (**b**) total ammonia concentration vs. stirring speed; (**c**) liquid/solid ratio vs. stirring speed; (**d**) total ammonia concentration vs. leaching time; (**e**) liquid/solid ratio vs. leaching time; (**f**) liquid/solid ratio vs. total ammonia concentration.

Based on the above regression analysis and variance analysis, the effects of different factors on the Zn effective extraction rate were investigated, by establishing 3D response surfaces of the regression model based on statistical calculations of the regression coefficients. Based on the optimised model, the response surfaces of the influences of stirring speed, leaching time, total ammonia concentration, and liquid/solid ratio, as well interactions between these factors on the Zn effective extraction rate, were obtained, and the results are plotted in Figure 4.

As illustrated in Figure 4a–c, leaching time, total ammonia concentration, and liquid/solid ratio presented greater effects on the Zn effective extraction rate than stirring speed. It can be observed in Figure 4d,e that the influences of total ammonia concentration and liquid/solid ratio showed greater effects than leaching time. Moreover, it is depicted in Figure 4f that the interactions between the total ammonia concentration and liquid/solid ratio exerted the most significant effects on the Zn effective extraction rate, and this conclusion is consistent with the above variance analysis. Furthermore, as shown in Figure 4f, simultaneously increasing total ammonia concentration and liquid/solid ratio can significantly improve the Zn effective extraction rate, before reaching a plateau value. The influences of total ammonia concentration and liquid/solid ratio are different, wherein increasing the total ammonia concentration increases the concentration of coordination agents, while the increase in L/S ratio can enhance the rapid dissolution of zinc oxide, and the solubility of [Zn(NH_3_)]^2+^ and (RCOO)_2_Zn in solution can be increased accordingly, further promoting the dissolution of zinc in MSD samples. As a result, the ion diffusion resistance decreases, and the leaching behaviour of Zn ion is strengthened, further enhancing the Zn effective extraction rate. However, once the values of the total ammonia concentration and liquid/solid ratio exceeded critical levels, the further increase in the two factors values had negligible effects on the Zn effective extraction rate. Since the materials in the container remain constant, the interfacial areas of Zn-containing minerals and NH_3_-CH_3_COONH_4_-H_2_O leaching agents are constant. Therefore, under the conditions of the sufficiently large quantity of the leaching agent and maintaining the effective mass transfer and maximal reactions between Zn-containing minerals, the Zn effective extraction rate can be maximised by leaching from the MSD sample using NH_3_-CH_3_COONH_4_-H_2_O leaching agent.

### 3.4. Condition Optimisation and Verification

Based on the predictions of the response surface methodology (RSM), the leaching time, total ammonia concentration, liquid/solid ratio, ammonia/ammonium ratio, stirring speed, and leaching temperature were optimised, and the results were experimentally verified. Table 8 lists the predicted values and experimental values of the Zn effective extraction rate achieved by coordination leaching from the MSD materials by NH_3_-CH_3_COONH_4_-H_2_O solution, as well as the optimised conditions and model verification results. Three leaching experiments based on the optimised process parameters were conducted to confirm the accuracy of the optimization parameters obtained by RSM. The average value of the Zn effective extraction rate in three parallel experiments was 84.67%; in contrast, the predicted value was 85.25%, demonstrating that RSM provides a reliable method to optimise the recovery process of Zn from Zn-containing MSD materials by coordination leaching using NH_3_-CH_3_COONH_4_-H_2_O solution.

### 3.5. Characterization Analysis

#### 3.5.1. XRD Analysis

**Figure 5:**
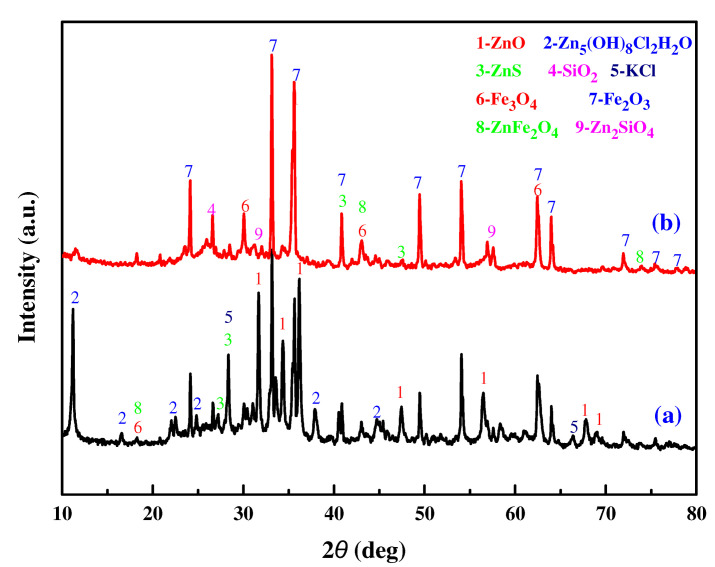
XRD pattern of the MSD sample (**a**) and leaching residue (**b**).

To determine the metal ions and impurities existing in the MSD sample and the leaching residues, the MSD samples and the leaching residues were characterized by a rotating target multi-functional X-ray diffractometer (TTRA III, Rigaku, Japan). The operation power of the X-ray generator was 18 kW, CuKα irradiation (λ = 1.54056 Å) was applied, and the voltage and the current were 40 kV and 200 mA, respectively. Under filtering using a graphite monochromator with a high reflection efficiency, scanning was conducted with a scanning rate of 4°/min from 10°–90°. The XRD pattern of the MSD sample is illustrated in Figure 5a. The XRD pattern revealed that Zn presented as ZnO, Zn_5_(OH)_8_Cl_2_·H_2_O, ZnS, ZnFe_2_O_4_, and Zn_2_SiO_4_, while Fe mainly presented as Fe_3_O_4_ and Fe_2_O_3_. The diversity of the zinc phase and the presence of iron suggest that the process of extracting zinc from the MSD sample is very difficult. Figure 5b shows an XRD comparison of leaching residues under optimized conditions. As shown in Figure 5, the diffraction peaks of ZnO and Zn_5_(OH)_8_Cl_2_H_2_O disappear, and the main residual zinc phases in the leaching slag are zinc ferrite (ZnFe_2_O_4_), zinc silicate (ZnSiO_4_), and zinc sulfide (ZnS).

#### 3.5.2. SEM-EDS Analysis

**Figure 6:**
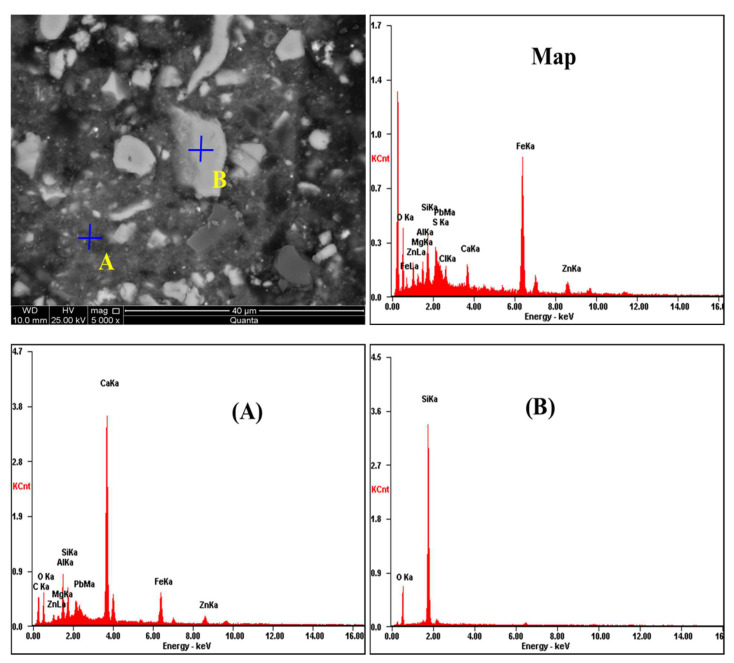
SEM-ESD spectra of the MSD sample.

**Figure 7:**
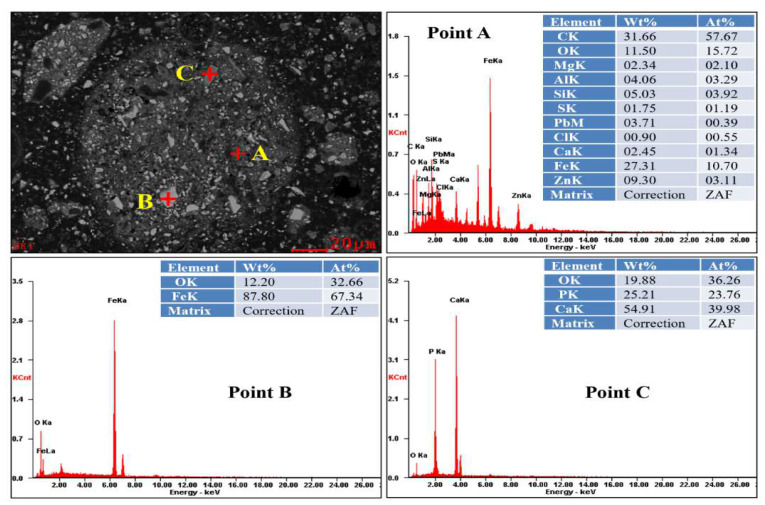
SEM-ESD pattern of MSD leaching residue.

To further investigate the particle distribution, morphology, and composition of the MSD sample and the leaching residue, the microstructures of the MSD sample and zinc leaching residue were determined using the SEM apparatus (XL30ESEM-TMP, Philips, The Netherlands), allocated with an EDS detector (EDS-Genesis, EDAX, Mahwah, NJ, USA), and the SEM-ESD spectra are displayed as shown in Figure 6 and Figure 7, respectively. Figure 6 shows that the grey flocculent amorphous structures in area A contained Zn, metals (Fe, Pb, and Al), and gangue components (Si, Ca, and Mg); the phase of area B was mainly quartz (SiO_2_). Moreover, metal inclusions and gangue components were observed. Figure 7 shows that the structural topography and particle element distribution of the residue, and the presence of C, O, Mg, Al, Si, S, Pb, Cl, Ca, Fe, P, and Zn, are relevant to the investigated system. There are three main morphologies in this sample: Point (A), dispersed flocculent structure particles; Point (B), bright grey massive structural particles; and Point (C), dark grey massive structural particles. The three structural particles are inlaid and tightly bound to each other.

**Figure 8:**
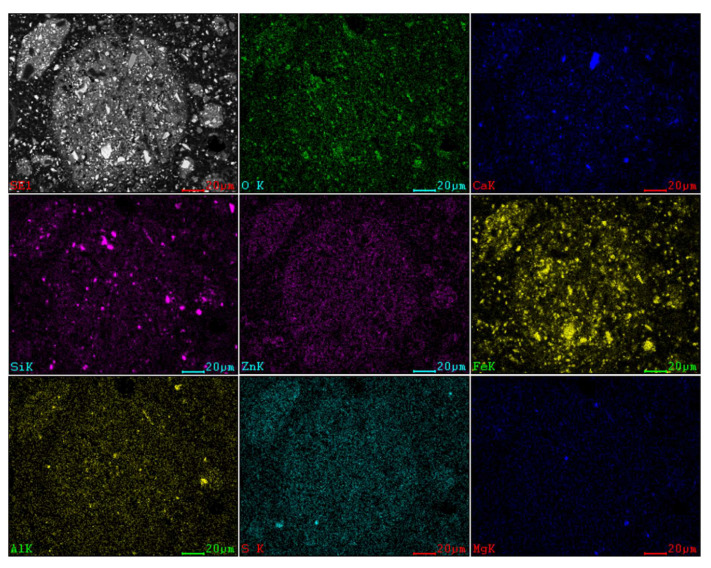
SEM-EDS map scanning pattern of MSD leaching residue.

The X-ray EDS maps of Cl, Al, Mg, Ca, O, Fe, Si, and Zn are analysed as shown in Figure 8. The EDS analysis of Point (A) shows that the dispersed flocculent structure particles are mainly composed of C, O, Mg, Al, Si, S, Pb, Cl, Ca, Fe, P, and Zn. The EDS analysis of Point (B) shows that the bright grey massive structural particles are mainly iron oxides. The EDS analysis of Point (C) shows that dark grey massive structural particles are mainly gangue minerals.

The SEM-EDS surface-scanning pattern shows that the residue comprises a metallic minerals phase in addition to a gangue minerals phase. Furthermore, the surface-scanning pattern makes it clear that Zn_2_SiO_4_, ZnS, and ZnFe_2_O_4_ do not leach in the NH_3_-CH_3_COONH_4_-H_2_O system.

## 4. Conclusions

In this study, Zn-containing metallurgical slag and dust (MSD) materials were utilised as the research object, and response surface methodology (RSM) was introduced into the process optimisation of recovering Zn from Zn-containing MSD materials by coordination leaching using NH_3_-CH_3_COONH_4_-H_2_O solution. The main findings were as follows:

(1) At a constant leaching temperature and ammonia/ammonium ratio, the influences of liquid/solid ratio, stirring speed, leaching time, total ammonia concentration, and interactions between them on the Zn effective extraction rate were investigated using the central composite design. The mathematic model of the factors affecting the Zn effective extraction rate was established as:
  Y = 79.19 + 0.38X_1_ + 0.84X_2_ + 8.55X_3_ + 7.25X_4_ − 0.012X_1_X_2_ + 0.10X_1_X_3_ + 0.28X_1_X_4_ −0.20X_2_X_3_ + 0.36X_2_X_4_ − 4.30X_3_X_4_ − 0.16X_1_^2^ − 0.69X_2_^2^ − 3.27X_3_^2^ − 3.34X_4_^2^

(2) The optimised parameters for the leaching experiments were obtained: the leaching temperature was 25 °C, the total ammonia concentration was 4.8 mol/L, the leaching time was 46 min, the liquid/solid ratio was 4.3:1, [NH_3_]/[NH_4_]^+^ was 1:1, the stirring speed was 345 r/min, the measured Zn effective extraction rate was 84.67%, and the Zn predicted effective extraction rate was 85.25%. The experimental and predicted values of the Zn effective extraction rate were similar, indicating the reliability of the proposed model and the suitability of the optimised process parameters.
(3) The XRD and SEM-EDS analysis results showed that Zn_2_SiO_4_, ZnS, and ZnFe_2_O_4_ were among the main factors affecting the low extraction rate of zinc from metallurgical slag dust under the CH_3_COONH_4_ system.

## Figures and Tables

**Table 1:** Chemical compositions of the MSD sample.

Compositions	Zn	Fe	C	Si	Cl	S
Mass (w%)	24.74	21.66	9.14	2.66	2.94	1.39
Compositions	Mg	Bi	Pb	In (g/t)		
Mass (w%)	1.14	0.97	1.13	354		

**Table 2:** Particle size distribution parameters of the MSD sample.

D_10_ (μm)	D_50_ (μm)	D_90_ (μm)	D_98_ (μm)	Volume Average Particle Size (μm)	Area Average Particle Size (μm)	Surface Area to Volume Ratio (m^2^/cm^3^)
2.47	7.89	24.85	47.91	10.78	5.59	6.42

**Table 3:** Response surface method factors’ level coding.

Factors	Levels		
	−1	0	1
Stirring speed (*X*_1_, r/min)	250	300	350
Leaching time (*X*_2_, min)	20	35	50
Total ammonia concentration (*X*_3_, mol/L)	3	4	5
Liquid/solid ratio (*X*_4_, mL/g)	3	4	5

**Table 4:** Centre composite design plan and Zn effective leaching rate results.

Number	*X*_1_ (r/min)	*X*_2_ (min)	*X*_3_ (mol/L)	*X*_4_ (L/S)	*Y* (%)
1	300	35	4	6	81.88
2	300	35	6	4	82.75
3	300	35	4	4	79.19
4	350	20	5	3	78.08
5	300	35	4	4	79.19
6	300	35	4	4	79.19
7	350	20	5	5	83.33
8	250	20	5	5	81.03
9	350	50	3	5	76.30
10	300	35	4	4	79.19
11	350	50	5	3	78.51
12	350	20	3	5	73.82
13	250	50	5	5	82.98
14	250	20	3	5	72.69
15	250	20	5	3	77.83
16	200	35	4	4	77.62
17	250	50	3	5	75.27
18	250	50	5	3	78.01
19	350	50	5	5	84.50
20	350	50	3	3	53.21
21	300	65	4	4	78.28
22	300	35	4	2	48.67
23	350	20	3	3	52.09
24	250	50	3	3	52.59
25	300	35	4	4	79.19
26	300	5	4	4	73.50
27	400	35	4	4	78.36
28	250	20	3	3	51.91
29	300	35	4	4	79.19
30	300	35	2	4	48.39

**Table 5:** Model fitting parameters for the designed experiments.

The Sequential Model Sum of Squares						
Source	Sum of Squares	df	Mean Square	*F* Value	*p*-Value Probd > *F*	
Mean vs. total	160,900	1	1.609 × 10^5^			
Linear vs. mean	3036.32	4	759.08	22.16	<0.0001	
2FI vs. linear	300.53	6	50.09	1.71	0.1726	
Quadratic vs. 2FI	536.69	4	134.17	104.59	<0.0001	Suggested
Cubic vs. quadratic	15.57	8	1.95	3.71	0.0505	Aliased
Residual	3.67	7	0.52			
Total	1.647 × 10^5^	30	5491.61			
Lack of fit tests						
Source	Sum of squares	df	Mean square			
Linear	856.46	20	42.82			
2FI	555.93	14	39.71			
Quadratic	19.24	10	1.92	Suggested		
Cubic	3.67	2	1.84	Aliased		
Pure error	0.00	5	0.00			
Model summary statistics						
Source	Std.		Adjusted R-squared	Predicted R-squared	PRESS	
	Dev.	R-squared				
Linear	5.85	0.7800	0.7448	0.6832	1233.10	
2FI	5.41	0.8572	0.7820	0.7741	879.23	
Quadratic	1.13	0.9951	0.9904	0.9715	110.84	Suggested
Cubic	0.72	0.9991	0.9961	0.8641	528.85	Aliased

**Table 6:** Credibility analysis for Zn effective leaching rate.

Std. Dev.	1.13	R-Squared	0.9951
Mean	73.22	Adj R-Squared	0.9904
C.V.%	1.55	Pred R-Squared	0.9715
PRESS	110.84	Adeq Precision	44.8820

**Table 7:** Variance analysis for response surface quadratic model.

Source	Sum of Squares	df	Mean Square	*F* Value	*p*-Value (Probd > *F*)	Significance
Model	3873.53	14	276.68	215.67	<0.0001	significant
*X*_1_	3.38	1	3.38	2.64	0.1252	
*X*_2_	16.92	1	16.92	13.19	0.0025	significant
*X*_3_	1752.92	1	1752.92	1366.39	<0.0001	significant
*X*_4_	1263.10	1	1263.10	984.57	<0.0001	significant
*X*_1_ *X*_2_	2.26 × 10^−3^	1	2.26 × 10^−3^	1.76 × 10^−3^	0.9671	
*X*_1_ *X*_3_	0.16	1	0.16	0.13	0.7273	
*X*_1_ *X*_4_	1.23	1	1.23	0.96	0.3437	
*X*_2_ *X*_3_	0.61	1	0.61	0.48	0.5002	
*X*_2_ *X*_4_	2.08	1	2.08	1.62	0.2222	
*X*_3_ *X*_4_	296.44	1	296.44	231.07	<0.0001	significant
*X*_1_^2^	0.72	1	0.72	0.56	0.4655	
*X*_2_^2^	12.94	1	12.94	10.09	0.0063	significant
*X*_3_^2^	292.75	1	292.75	228.20	<0.0001	significant
*X*_4_^2^	306.12	1	306.12	238.61	<0.0001	significant
Residual	19.24	15	1.28			
Lack of Fit	19.24	10	1.92			
Pure Error	0	5	0			
Cor Total	3892.78	29				

**Table 8:** The optimised process parameters determined by the regression model.

Total Ammonia Concentration (mol/L)	[NH_3_]/[NH_4_]^+^ Mole Ratio	Temperature (°C)	Time (min)
4.78	1:01	25	46.20
Liquid/solid ratio (mL/g)	Stirring speed (r/min)	Zn effective leaching rate (%)	
		Predicted value	Experimental value
4.29	344.78	85.25	84.67

## Data Availability

The data presented in this study are available on request from the corresponding author. The data are not publicly available due to technical or time limitations.
